# Inherited STAT1 Deficiency in a Child with BCG-osis and Severe COVID-19 Pneumonia

**DOI:** 10.1007/s10875-023-01510-x

**Published:** 2023-06-01

**Authors:** Mame Sokhna Guèye, Mame Téné Ndiaye-Diop, MSMD Consortium, Tom Le Voyer, Camille Soudée, Idrissa Demba Ba, Awa Kane, Indou Dème-Ly, Joséphine Khady Badiane-Seye, Anne-Sophie L’Honneur, Abdoul Aziz Diallo, Ousmane Ndiaye, Macoura Gadji, Qian Zhang, Souleymane Mboup, Jean-Laurent Casanova, Jacinta Bustamante, Tandakha Ndiaye Dièye

**Affiliations:** 1 Institute for Health Research, Epidemiological Surveillance and Training (IRESSEF), Dakar, Senegal; 2 Albert Royer National Children’s Hospital Center, Dakar, Senegal; 3 Dermatology Department, Cheikh Anta Diop University, Dakar, Senegal; 4 Paris Cité University, Imagine Institute, Paris, France; 5 Laboratory of Human Genetics of Infectious Diseases, Necker Branch, INSERM U1163, Necker Hospital for Sick Children, Paris, France; 6 St. Giles Laboratory of Human Genetics of Infectious Diseases, Rockefeller Branch, The Rockefeller University, New York, NY USA; 7 Study Center for Primary Immunodeficiencies, Necker Hospital for Sick Children, Assistance Publique-Hôpitaux de Paris AP-HP, Paris, France; 8 National Blood Transfusion Center, Dakar, Senegal; 9 Immunology Department, Cheikh Anta Diop University, Dakar, Senegal; 10 Department of Virology, Paris Cité University and Cochin Hospital, Paris, France; 11 Department of Pediatrics, Necker Hospital for Sick Children, AP-HP, Paris, France; 12 Howard Hughes, Medical Institute, New York, NY USA

## To the Editor,

The human signal transducer and activator of transcription 1 (STAT1) protein is one of seven members of the STAT family. STAT1 was the first member identified as a key molecule required for cellular responses to type I, II, and III interferons (IFNs) [1]. Germline variants in human *STAT1* cause four types of inborn errors of immunity (IEIs): (i) autosomal recessive (AR) complete STAT1 deficiency, (ii) AR partial STAT1 deficiency, (iii) autosomal dominant (AD) STAT1 deficiency, and (iv) AD STAT1 gain-of-function (GOF) [2]. AR partial STAT1 deficiency underlies syndromic Mendelian susceptibility to mycobacterial disease (MSMD), predisposing otherwise healthy individuals to infections caused by weakly virulent mycobacteria, such as the *Bacille Calmette-Guérin* (BCG) vaccine strain and environmental mycobacteria (EM). These patients also suffer from severe viral infectious diseases. AR partial STAT1 deficiency is caused by decreased STAT1 protein expression, resulting in less severe symptoms than in patients with AR complete STAT1 deficiency [2]. Here, we report a new patient with AR partial STAT1 deficiency, with a history of BCG-osis and COVID-19 pneumonia.

**Fig. 1:**
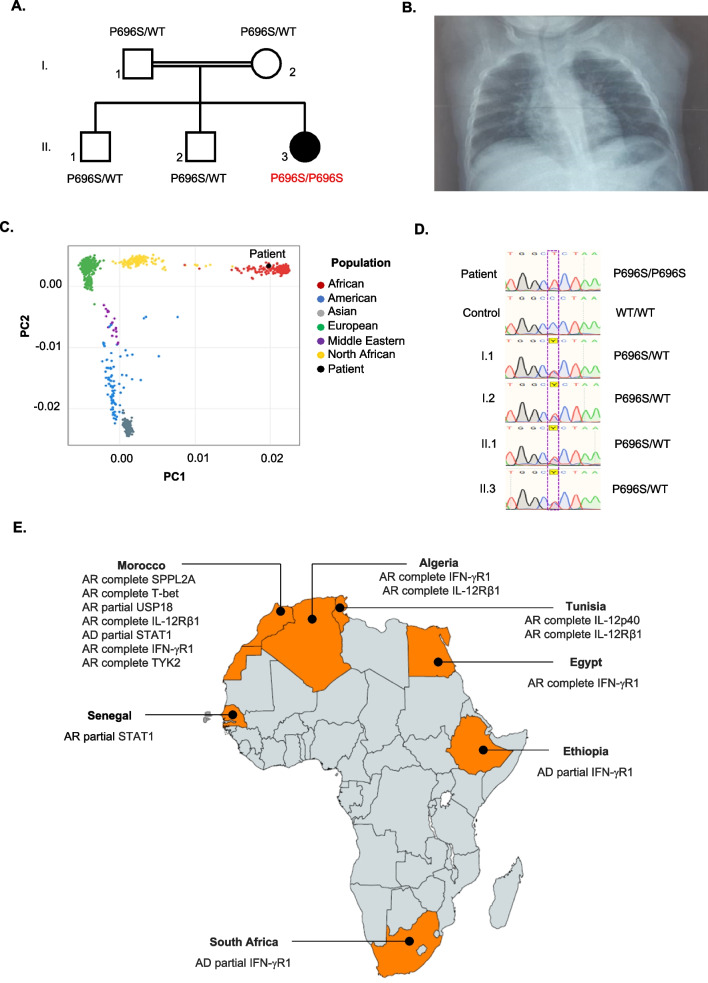
Autosomal recessive partial STAT1 deficiency in Senegal. (**A**) *STAT1* genotype and pedigree of the kindred. The index case is indicated with a black circle. (**B**) Patient’s chest X-rays (showing a bilateral interstitial pneumonia). (**C**) Patient’s ethnicity by principal component analysis (PCA). (**D**) Genomic sequences in the sense orientation of exon 23 of *STAT1* (NM_001384891.1) in the index patient, a healthy control and the other family members as indicate in the pedigree. (**E**) Map of the African countries where MSMD cases have been identified (orange colored countries) and their respective diseases

The index patient was a 5 month-old female infant born at term to second-degree consanguineous parents (Fig. 1A) from Dakar recruited at the Albert Royer National Children Hospital Center in 2020. She had no antenatal or perinatal difficulties and no history of IEI. She received all vaccines following the Senegalese expanded immunization program. However, she did not receive COVID-19 vaccine. A few weeks after the BCG vaccination, the patient presented an enlarged left periaxillary lymph node (BCG-itis) and lung involvement (pneumonia), accompanied by serous rhinitis. A Genexpert MTB test of the bronchoalveolar fluid was negative for *M. tuberculosis* complex. Routine blood tests showed hyperleukocytosis (40.17 G/L neutrophils—normal range (NR): 1.7–7 G/L; 10.80 G/L lymphocytes—NR: 2–4 G/L; 5.13 G/L monocytes—NR: 0.4–1 G/L), an increase of C reactive protein (CRP: 404.5 mg/L—NR : 0–6 mg/L) and hyper alpha and gammaglobulinemia with high IgG and IgM rates (alpha 1 globulin 9.1 g/L—NR: 2.1–3.5 g/L; gamma globulin 18.0 g/L—NR: 8.0–13.5 g/L). Chest X-rays showed bilateral lung disease (Fig. 1B). The histological examination of skin biopsies undertaken at the axillary lymph node revealed the presence of tuberculoid granulomas, compatible with cutaneous mycobacterial infection. Treatment was initiated with rifampicin, isoniazid, and ethambutol for two months followed by rifampicin and isoniazid for six months.

During her follow-up, she suffered two episodes of severe pneumopathy, the last one in 2021 during the COVID-19 pandemic. The patient developed an acute respiratory distress, leading to hospitalization and intensive care unit admission. Oxygen therapy at 3 L/min was implemented for 24 hours to correct oxygen desaturation, along with a high-calorie diet and vitamin therapy. A chest computed tomography (CT) scan identified bilateral interstitial pneumonia. She received steroids, salbutamol, and antibiotic therapies (ceftriaxone, vancomycin, and amikacin) with good response after 15 days of treatment. A PCR for COVID-19, performed during hospital admission, was negative; however, positive SARS-CoV2 immunoglobulins were detected eight months later. Additionally, viral serologies were high for cytomegalovirus and Epstein Barr virus immunoglobulins whereas Herpes simplex viruses (HSV) 1 and 2 IgG were within normal range (Supplemental Table). Autoantibodies against IFN-α2 test were negative. The patient had good psychomotor development and no similar cases were reported in the family.

Due to the severity of the infections, a diagnosis of IEI was suspected and genetic testing was carried out by whole-exome sequencing (WES). The child’s ethnicity was confirmed by principal component analysis (PCA) (Fig. 1C). WES revealed a homozygous variant in exon 23 of *STAT1* (NM_001384891.1) corresponding to a substitution of cytosine by thymine at position 2086 (c.2086C > T) and leading to a change of proline to serine at position 696 (p.P696S) in the protein. Sanger sequencing confirmed homozygosity for the variant in the index patient, and the other family members were heterozygous (Fig. 1D). The p.P696S variant in this patient was previously reported to underlie AR partial STAT1 deficiency and is located between the SH2 and tail segment domains of STAT1, resulting in reduced STAT1 expression and decreased responses to both IFN-α and IFN-γ. Our findings strongly suggest that homozygosity for p.P696S underlies the clinical disease in this patient [2].

Since 2014, Senegal has been conducting studies to identify IEIs in the country, with most cases suffering from severe combined immunodeficiencies (SCID), combined immunodeficiencies (CID) with associated or syndromic features, and neutropenia. However, no case of MSMD has been described in Senegal so far.

This patient has MSMD due to AR partial STAT1 deficiency and presented with two severe infectious diseases caused by *M. bovis* BCG and SARS-CoV2. In Africa, cases of MSMD (Fig. 1E) have been reported in Algeria, Egypt, Ethiopia, Morocco, South Africa, and Tunisia (https://www.asid-africa.org/en/). This is the first case of MSMD in Senegal and Sub-Saharan countries. We propose MSMD in the differential diagnosis of children with BCG related diseases (or even tuberculosis). Furthermore, as BCG vaccination is mandatory in infants at birth in Senegal, it should be delayed in siblings of affected children until they get tested.

IEIs affecting type I IFN immunity confers a predisposition to life-threatening COVID-19 pneumonia [3]. Recessive or dominant deficiencies have been reported in unvaccinated adults with critical COVID-19 pneumonia. More recently, Zhang et al. reported the first international cohort of 112 children in which they found recessive complete deficiencies (TLR7, IFNAR1, STAT2, or TYK2) in around 4% of children with critical or severe COVID-19 pneumonia [4]. AR STAT1 deficiency was predicted to confer susceptibility to COVID-19 pneumonia as reported patients with this IEI developed pneumonia caused by other viruses [2] due to unresponsiveness to both type I and III IFNs. Moreover, human deficient STAT1-/- SV-40 fibroblasts fail to control SARS-CoV2 replication after pre-treatment with type I IFN suggesting a greater susceptibility to this virus [5]. To our knowledge, this patient is the first with AR STAT1 deficiency associated with severe COVID-19 pneumonia. The favorable outcome of the SARS-CoV2 infection here may be the result of residual STAT-dependent cellular response to type I/III IFNs. Probably, this IEI is underdiagnosed in the context of severe viral pneumonia. Prompt identification of the genetic cause underlying the infectious phenotype in this patient is critical for genetic counseling in affected families and the medical treatment.

## Supplementary Information

ESM 1

## Data Availability

All data are either included in the manuscript or are available upon request.

## References

[1] Mizoguchi Y, Okada S (2021). Inborn errors of STAT1 immunity. Curr Opin Immunol..

[2] Le Voyer T, Sakata S, Tsumura M, Khan T, Esteve-Sole A, Al-Saud BK (2021). Genetic, immunological, and clinical features of 32 patients with autosomal recessive STAT1 deficiency. J Immunol..

[3] Zhang Q, Bastard P, Liu Z, Le Pen J, Moncada-Velez M, Chen J (2020). Inborn errors of type I IFN immunity in patients with life-threatening COVID-19. Science..

[4] Zhang Q, Matuozzo D, Le Pen J, Lee D, Moens L, Asano T (2022). Recessive inborn errors of type I IFN immunity in children with COVID-19 pneumonia. J Exp Med..

[5] Rosain J, Neehus A-L, Manry J, Yang R, Le Pen J, Daher W (2023). Human IRF1 governs macrophagic IFN-γ immunity to mycobacteria. Cell..

