# Supplementary material for: Inherited STAT1 Deficiency in a Child with BCG-osis and Severe COVID-19 Pneumonia
Source: J Clin Immunol. 2023 Jun 1;43(7):1479–82. doi: 10.1007/s10875-023-01510-x (PMC10232337; doi:10.1007/s10875-023-01510-x)
Supplement: Supplementary file 1 — ESM 1 [file 10875_2023_1510_MOESM1_ESM.docx]

**Supplemental Table:** Patient’s viral serology

| **Parameters** | | **Patient (2 y.o.)** | **Threshold** | **Interpretation** |
| --- | --- | --- | --- | --- |
| Cytomegalovirus | IgM anti-CMV | 26.4 | ≥ 22 | Positive |
|  | IgG anti-CMV | 180 | ≥ 14 | Positive |
|  | Avidity of IgG anti-CMV | 56.4 | ≥ 25 | Elevated |
| Epstein-Barr virus | IgM anti-VCA | < 10 | ≥ 40 | Negative |
|  | IgG anti-VCA | > 750 | ≥ 20 | Positive |
|  | IgG anti-EBNA | 137 | ≥ 20 | Positive |
| SARS-CoV2 | IgG anti-SARS-CoV2 | 926 | ≥ 33.8 | Positive |
| Rubella | IgG anti-rubella | < 5 | ≥ 11 | Negative |
|  | IgM anti-rubella | 37.4 | ≥ 25 | Positive |
| Measles virus | IgG anti-measles | < 5.00 | ≥ 16.5 | Negative |
|  | IgM anti-measles | 0.089 | ≥ 1.1 | Negative |
| Mumps virus | IgG anti-mumps | < 5.00 | ≥ 11 | Negative |
|  | IgM anti-mumps | 0.3 | ≥ 1.1 | Negative |
| Varicella Zoster Virus | IgG anti-VZV | 17.3 | > 165 | Negative |
| Herpes Simplex Virus 1 | IgG anti-HSV-1 | 0.0851 | ≥ 1.1 | Negative |
| Herpes Simplex Virus 2 | IgG anti-HSV-2 | < 0.500 | ≥ 1.1 | Negative |
| Hepatitis A Virus | IgG anti-HAV | 0.44 | ≥ 1 | Negative |
| Parvovirus B19 | IgG anti-Parvovirus B19 | < 0.10 | ≥ 1.1 | Negative |
